# Is the Morriston Occupational Therapy Outcome Measure (MOTOM) an appropriate tool for reablement services?

**DOI:** 10.1177/03080226241269255

**Published:** 2024-08-06

**Authors:** Sophie Tooke, Julia Warrener, Tamsin Leah, Joanna Ward, Jeremy Dearling

**Affiliations:** 1 Senior Reablement Occupational Therapist, Norfolk First Support, Norfolk County Council, Norwich, UK; 2 Deputy Dean, School of Health & Social Work, University of Hertfordshire, Hatfield, UK; 3 Occupational Therapist, Older Persons Physical Disability Team, Norfolk County Council, Norwich, UK; 4 Senior Occupational Therapist, Prevention and Early Intervention, Cambridgeshire County Council, Peterborough, UK; 5 Shaping Our Lives, King’s Lynn, UK

**Keywords:** Morriston occupational therapy outcome measure, reablement, social care, n05 – health care, V04 – research

## Abstract

**Introduction:**

The Care Act (2014) requires local authorities to provide reablement services but does not standardise how to do this, leading to different services utilising different outcome measures. This article investigates the Morriston Occupational Therapy Outcome Measure, which has been under researched in community reablement settings.

**Method:**

A questionnaire was distributed to the staff working within one local authority to seek their experience of using the Morriston Occupational Therapy Outcome Measure. The questionnaire consisted of closed and open-ended questions to gain insights into their understanding and experience of the Morriston Occupational Therapy Outcome Measure.

**Findings:**

Quantitative findings showed that staff felt they understood the Morriston Occupational Therapy Outcome Measure, and most respondents agreed that the Morriston Occupational Therapy Outcome Measure was an effective tool for reablement services. However, staff provided contradictory responses as to whether the Morriston Occupational Therapy Outcome Measure was applied consistently or that service users understand the assessment.

**Qualitative:**

Findings showed the Morriston Occupational Therapy Outcome Measure is a service user tool, service provider tool, and it provides quality assurance. However, the Morriston Occupational Therapy Outcome Measure can have restricted applicability and within this local authority, more training was needed to improve the consistency of goal-scoring.

**Conclusion:**

The Morriston Occupational Therapy Outcome Measure does have strengths within reablement services; however, to ensure it is an effective tool, this research highlights the need for a high level of training.

## Introduction

Reablement is defined as ‘services that help people live independently, provided in the person’s own home by a team of mainly social care professionals’ (Department of Health and Social Care, 2022a: 15). Reablement services are expected to increase in demand as the population continues to grow. Although reablement is not age-specific, it is understood that older people tend to use these services more than younger people (Mulquiny and Oakman, 2021). In the 2021 Census, there were 10.4 million people aged 65 and over in England (Office for National Statistics, 2022a). This population group is projected to increase by almost a third in the next 20 years in the United Kingdom (Office for National Statistics, 2022b). As a result, it is likely that the demand for services will increase, therefore identifying an appropriate outcome measure will enable services to increase efficacy and effectiveness.

The Social Care Reform states social care needs to ‘intervene early’ and ‘focus on prevention’ (Department of Health and Social Care, 2022b: 14–33). The National Health Service (NHS) specifies within the Long-Term Plan ‘all parts of the country should be delivering reablement’ (NHS, 2019: 14). In addition, the Care Act (2014) requires local authorities to provide or arrange for the provision of services that prevent the need for care and support. The Care Act statutory guidance includes reablement as an example of this type of service; however, it does not dictate how local authorities should implement this. The National Institute for Health and Care Excellence (NICE) have produced the Intermediate Care Including Reablement Guidelines (NICE, 2017), advising services what reablement should consist of. The guidelines state that reablement should be person-centred and practitioners should work in partnership with people to develop collaborative goals. It is important to consider whether the outcome measures chosen in services could facilitate or restrict the delivery of guidelines in practice. This research investigates one particular outcome measure, the Morriston Occupational Therapy Outcome Measure (MOTOM; James and Coor, 2004), to see if this is appropriate for reablement services and facilitates the delivery of guidelines or policy in practice.

## Aims

- Is the MOTOM an appropriate outcome measure for reablement services.
- Does the MOTOM facilitate the delivery of guidelines or policy in practice.

## Literature review

A literature search revealed that different local authorities utilise different outcome measures in reablement services such as the Canadian Occupational Performance Measure (COPM) (Hjelle et al., 2017a, 2017b). However, Beresford et al (2019a) found that some services do not use standardised measures to monitor outcomes at all. Whitehead et al (2016: 8) stated that ‘the National Audit for Intermediate Care has also previously reported much debate when agreeing which outcome measure to use for home-based intermediate care and reablement services’. This may be because reablement services vary in criteria, provision and delivery models (Beresford et al, 2019b; Feining et al., 2023). Although this variation exists, Parsons et al. (2012) have shown that the use of a goal-facilitation tool improves outcomes for service users. The COPM, along with other measures, such as TARGET goal setting (Whitehead et al., 2016), have been shown to require accurate scoring from practitioners to achieve reliability (Fuller, 2011). In addition, to enable successful goal setting, staff need to understand their role (Golenko et al., 2021). Although some outcome measures have been evaluated, there is no current literature on the use of the MOTOM in reablement settings.

The MOTOM was created in the Morriston Hospital in Swansea by Sharon James and Susan Coor in 1993 to be used alongside the Model of Adaptation through Occupation (Reed and Sanderson, 1992). James and Coor identified that a tool was needed to measure changes in occupational performance as a result of occupational therapy. The MOTOM is a goal facilitation tool that includes four stages: identifying the goal, the barriers to achieving the goal, the plan to overcome the barriers and a pre- and post-scoring of the goal that reflects the occupational therapy intervention. The MOTOM manual states goals ‘may relate to self-care, productivity or leisure’ (James and Coor, 2006: 5), and that only the goals the person wants and needs to do are included. Although reablement services exist internationally, it is not clear if the MOTOM is used internationally. However, the MOTOM manual advises the tool can be used in ‘most clinical settings’. This project aims to assess the suitability of the MOTOM in a community reablement setting.

## Method

To answer the research question, and assess whether the MOTOM is appropriate for reablement services, staff working within a reablement setting in one local authority were asked to complete a questionnaire on their experience of using the MOTOM. This local authority has been using the MOTOM since January 2021. The reablement service consists of service leads (SLs), service managers (SMs), practice consultant occupational therapists (PCOTs), occupational therapists (OTs), operational managers (OMs), reablement assistant practitioners (RAPs) and reablement support workers (RSWs). A questionnaire was selected as the method of collecting data from service providers with limited time to engage in research. In addition, a questionnaire was a time efficient way for the research team to reach more staff groups which was seen as important due to the variation of staff that were using the MOTOM. The objective was to achieve a representative response rate and ensure each staff member had a fair and equal chance to complete the questionnaire. It was decided two questionnaires would be created, one for SLs, SMs, PCOTs, OTs, OMs and RAPs as these staff either write or authorise the assessment. The other questionnaire was for RSWs who work directly with the service user to meet their reablement goals and measure outcomes. The questionnaires were designed slightly differently due to the differences in the service provider’s role.

The research team included four occupational therapists working in a variety of teams within social care services and two people that have lived experience of social care services. The research team worked together to design the questionnaires. A pilot study was completed with each professional group and changes were made to improve the clarity of questions. Questionnaire 1 (Q1) consisted of 18 questions and Questionnaire 2 (Q2) consisted of 17 questions. The questionnaire was designed so that the participant could complete it within 30 minutes and contained closed and open-ended questions. For the closed-ended questions, demographic data, including professional role, area of service provision and how long the staff member had been in the service was obtained. The participant had to state the degree to which they agreed or disagreed with a series of statements on a sliding scale. The scale consisted of five options from strongly disagree to strongly agree. The open-ended questions were included to obtain more information from the participant about how and why they felt the way they did about the MOTOM that was difficult to get from the closed-ended questions alone. For this reason, thematic analysis was used due to the reflexive nature of this process. Thematic analysis consists of six phases: familiarisation of the data set, coding, generating initial themes, developing and reviewing themes, refining, defining and naming themes and writing-up (Braun & Clarke, 2022). The research lead completed all six steps of thematic analysis for consistency and, to reduce bias, every question was also analysed by a member of the research team so that collectively the team could discuss, refine and define the themes found.

Ethical approval was obtained from a UK university faculty ethics committee. Research governance was also obtained from the local authority.

All participants were invited to complete the online questionnaire, through Jisc Surveys, which was open for 1 month (June 2023). A reminder to complete the questionnaire was sent half-way through the data collection period and the research lead attended team meetings to encourage responses.

Q1 was sent to 100 staff and 54 provided consent. Q2 was sent to 305 staff and 99 provided consent. In total, 153 staff completed the questionnaires (a 37.7% response rate overall). The findings of both questionnaires will be explored in further detail.

## Findings

### Quantitative findings

#### Questionnaire 1

**Figure 1.**
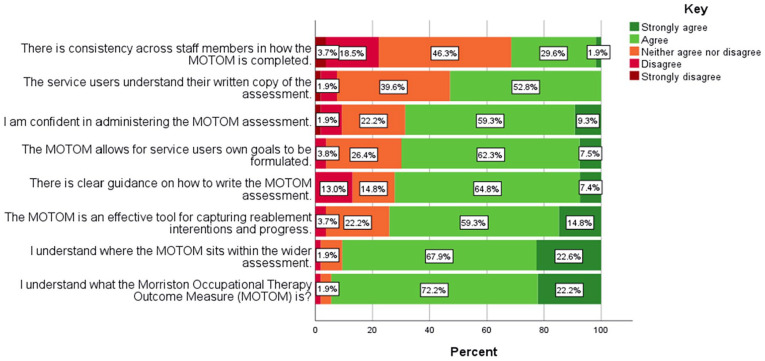
Questionnaire 1 Likert Scale. *N* = 54.

The questionnaire was completed by SLs, SMs, PCOTs (21.2%), OTs (7.7%), OMs (34.6%) and RAPs (36.5%). These distributions are reflective of the service structure. The respondents were from different geographical areas of service provision within one local authority and 53.6% of staff had at least 4 years of experience in the service. The results of the questionnaire can be seen in Table 1 and Figure 1.

**Table 1.** Questionnaire 1 results.

Question	Response	*n* (%)
What is your role?	Service Lead/Service Manager/Practice Consultant Occupational Therapist	11 (21.2)
	Reablement Occupational Therapist	4 (7.7)
	Operational Manager	18 (34.6)
	Reablement Assistant Practitioner	19 (36.5)
What area are you allocated to in the service?	City Centre	14 (25.9)
	North	12 (22.2)
	South	12 (22.2)
	West	7 (13)
	East	7 (13)
	Not Applicable	2 (3.7)
How long have you worked within the service?	Less than 1 year	11 (20.4)
	2–3 years	13 (24.1)
	4–5 years	7 (13.0)
	Over 5 years	23 (42.6)
The average time it takes you to complete an initial assessment with a service user:	Up to 1 hour	10 (19.6)
	Up to 2 hours	36 (70.6)
	Up to 3 hours	5 (9.8)
	Up to 4 hours	0 (0)
The average time it takes you to type-up the assessment:	Up to 1 hour	36 (75)
	Up to 2 hours	11 (22.9)
	Up to 3 hours	1 (2.1)
	Up to 4 hours	0 (0)
The most common types of goals set using the MOTOM are:	Self-care (brushing teeth, washing and dressing)	50 (92.6)
	Productivity (housekeeping, preparing meals)	2 (3.7)
	Leisure (accessing the community, social events)	1 (1.9)
	Other	1 (1.9)
Is there anything you would improve, include or remove in the reablement assessment?	Yes	18 (34.6)
	No	34 (65.4)

MOTOM: Morriston Occupational Therapy Outcome Measure.

*N* = 54.

The statements that had the highest proportions of agreement were that staff ‘understood what the MOTOM is’, and ‘where this outcome measure sits within the wider reablement assessment’. On the other hand, the statements that had higher proportions of disagreement were that ‘there is consistency across staff members in how the MOTOM is completed’, and that ‘there is clear guidance on how to write the MOTOM assessment’.

An additional key finding is that most staff within Q1 (92.6%) reported that self-care was ‘the most common type of goal set using the MOTOM’ compared with productivity (3.7%), leisure (1.85%) or other (1.85%) goals. Furthermore, most staff (65.4%) reported that they would not improve the assessment.

The answers to the questions were cross-tabulated with the demographic variables including the service provider’s professional role, the area of service provision and the years of service provision. Non-parametric statistical tests were completed to test if the results were statistically significant at the 95% confidence level (see Table 2).

**Table 2.** Questionnaire 1 Fishers Exact tests.

Question	Demographic	Fisher’s exact	*p* Value
I understand what the Morriston Occupational Therapy Outcome Measure (MOTOM) is.	Role	13.997	0.023*
	Area	17.464	0.237
	Length of time	6.549	0.793
I understand where the MOTOM sits within the wider reablement assessment.	Role	10.539	0.052
	Area	20.335	0.067
	Length of time	8.874	0.402
There is clear guidance on how to write the MOTOM assessment.	Role	9.027	0.352
	Area	11.764	0.686
	Length of time	11.709	0.128
I am confident in administering the MOTOM assessment.	Role	11.786	0.420
	Area	19.032	0.536
	Length of time	17.388	0.043*
The MOTOM is an effective tool for capturing reablement interventions and progress.	Role	14.672	0.041*
	Area	11.329	0.794
	Length of time	7.412	0.580
There is consistency across staff members in how the MOTOM is completed.	Role	8.117	0.966
	Area	21.475	0.342
	Length of time	17.637	0.049*
The most common types of goals set using the MOTOM.	Role	10.481	0.252
	Area	18.224	0.202
	Length of time	9.578	0.242
The average time it takes you to complete an initial assessment with a service user.	Role	15.991	0.003*
	Area	11.916	0.213
	Length of time	5.483	0.455
The average time it takes you to type-up the assessment.	Role	10.987	0.039*
	Area	11.912	0.357
	Length of time	4.422	0.757
The MOTOM allows for the service user’s own goals to be formulated.	Role	20.05	0.002*
	Area	12.993	0.600
	Length of time	6.955	0.638
The service users understand their written copy of the assessment.	Role	8.464	0.142
	Area	13.025	0.783
	Length of time	16.69	0.013*
Is there anything you would improve, include or remove in the reablement assessment?	Role	1.966	0.639
	Area	4.783	0.456
	Length of time	1.828	0.644

*N* = 54, *p < 0.05.

There was a statistically significant difference in understanding the MOTOM across different professional roles (*p* < 0.023). Although 94.4% of the respondents in Q1 agreed with the statement ‘I understand what the MOTOM is’, there were higher proportions of OMs (5.6%) and RAPs (5%) that selected ‘neither agree nor disagree’ compared with 0% of occupational therapists, PCOTs, SMs and SLs.

There is a variation in the degree of confidence staff have in administrating the MOTOM across different levels of time spent in the service. This variation is statistically significant at the 5% level (*p* < 0.043). From looking at this association, it does not appear that confidence with the MOTOM increases with experience. In fact, 36.36% agreed they felt confident after being in the service for up to 1 year, 92.3% up to 2 years, 100% up to 3 years and 60.87% for staff that had been in the service for over 5 years. However, it is important to note that the MOTOM has only been used in the service for 2 years when staff completed this questionnaire.

There was also a statistically significant difference between staff thinking ‘the MOTOM allows for service users own goals to be formulated’ across different professional roles (*p* < 0.002). A total of 100% of SLs, SMs, and PCOTs ‘agreed’ or ‘strongly agreed’ with this statement contrasting with 100% of occupational therapists that ‘neither agreed nor disagreed’. In addition, 10.5% of RAPs selected ‘disagree’ that ‘the MOTOM allows for the service users own goals to be formulated’. The RAPs are the staff members that complete the MOTOM with every service user within the service. It is an interesting finding to see the staff that set the most goals were the only professional group to disagree with this statement.

#### Questionnaire 2

**Figure 2.**
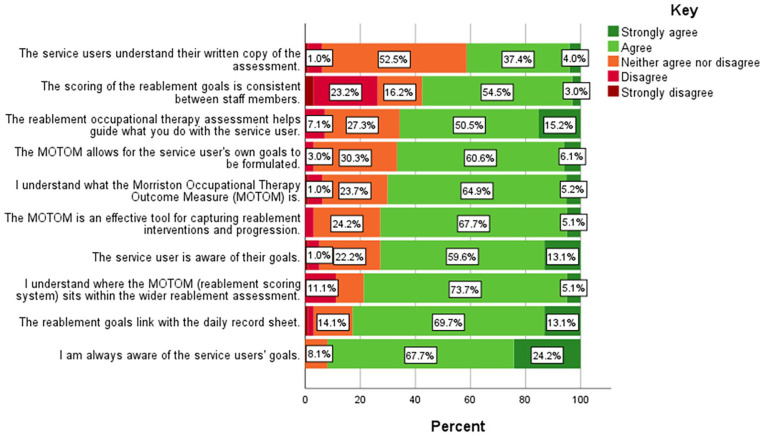
Questionnaire 2 Likert Scale. *N* = 99.

This questionnaire was completed by RSWs, please refer to Table 3 and Figure 2 for the findings. The staff were distributed geographically across the service areas and 67.7% of respondents have been in the service for at least 4 years.

**Table 3.** Questionnaire 2 results.

Question	Response	*n* (%)
What area are you allocated to in the service?	City Centre	26 (26.3)
	North	18 (18.2)
	South	13 (13.1)
	West	29 (29.3)
	East	13 (13.1)
How long have you worked within the service?	Less than 1 year	8 (8.1)
	2–3 years	24 (24.2)
	4–5 years	15 (15.2)
	Over 5 years	52 (52.5)
The most common types of goals set using the MOTOM are:	Self-care (brushing teeth, washing, dressing)	92 (94.8)
	Productivity (housekeeping, preparing meals)	2 (2.1)
	Leisure (accessing the community, social events)	0 (0)
	Other	3 (3.1)
Is there anything you would improve, include or remove in the reablement assessment?	Yes	39 (41.5)
	No	55 (58.5)

MOTOM: Morriston Occupational Therapy Outcome Measure.

*N* = 99.

The majority of staff within Q2 (94.85%) also felt self-care was ‘the most common types of goal set using the MOTOM’ compared with productivity (2.06%), leisure (0%) and other (3.09%) goals. In addition, 58.5% of Q2 respondents also reported that there was not anything they would ‘improve, include or remove in the reablement assessment’.

Similarly to Q1, majority of Q2 staff also agreed with most of the questionnaire statements as can be seen in Figure 2. The statements that had the highest proportions of ‘strongly agree’ and ‘agree’ were that staff were ‘aware of the service users’ goals’ and that ‘the reablement goals link with the daily record sheet’. In contrast, the statements that had higher proportions of ‘strongly disagree’ and ‘disagree’ were that ‘the scoring of the reablement goals is consistent between staff members’ and that they ‘understand where the MOTOM sits within the wider reablement assessment’.

The answers were cross-tabulated with demographic data including the service provider’s area and the years of service provision. Non-parametric statistical tests were completed to test if the results were statistically significant at the 95% confidence level (see Table 4).

**Table 4.** Questionnaire 2 Fishers Exact Tests.

Question	Demographic	Fisher’s exact	*p* Value
I understand what the Morriston Occupational Therapy Outcome Measure (MOTOM) is.	Area	19.370	0.113
	Length of time	13.812	0.228
I understand where the MOTOM sits within the wider reablement assessment.	Area	12.011	0.338
	Length of time	16.467	0.020*
The scoring of the reablement goals is consistent between staff members.	Area	25.486	0.012*
	Length of time	10.46	0.504
The MOTOM is an effective tool for capturing reablement interventions and progress.	Area	8.55	0.720
	Length of time	6.318	0.671
I am always aware of the service users’ goals.	Area	4.425	0.843
	Length of time	4.044	0.665
The most common types of goals set using the MOTOM.	Area	4.973	0.935
	Length of time	2.654	0.942
The service user is aware of their goals.	Area	13.319	0.663
	Length of time	9.715	0.673
The MOTOM allows for the service users’ own goals to be formulated.	Area	12.578	0.266
	Length of time	4.011	0.938
The service users understand their written copy of the assessment.	Area	21.383	0.051
	Length of time	8.243	0.825
The reablement occupational therapy assessment helps guide what you do with the service user.	Area	6.116	0.933
	Length of time	11.167	0.203
The reablement goals link with the daily record sheet.	Area	17.105	0.267
	Length of time	11.137	0.518
Is there anything you would improve, include or remove in the reablement assessment?	Area	5.491	0.241
	Length of time	1.848	0.622

*N* = 99, *p < 0.05.

There was a statistically significant difference in understanding where the MOTOM sits within the wider reablement assessment across the length of time the service provider has been in the service (*p* < 0.020). A total of 12.5% of Q2 respondents who have been in the service less than 1 year ‘disagree’ that they understand where the MOTOM sits within the wider assessment. Although we may expect this figure to be lower for staff with more experience, the proportion of staff that selected ‘disagree’ increased to 33.3% for those that have been in the service for 4-5 years. This suggests that there is a lack of clarity around the MOTOM regardless of experience levels.

A further statistically significant finding from Q2, is that the extent to which staff agree or disagree that ‘the scoring of the reablement goals is consistent between staff members’ varies dependent on the area of service provision (*p* < 0.012). For example, when looking at staff that had selected ‘agree’ or ‘strongly agree’ this varied across the geographical areas; South 30.77%, West 44.84%, North 55.6%, Central 73.08%, East 84.62%. This variation indicates mixed staff views on whether the MOTOM scoring is consistent.

A final statistical test, the Mann–Whitney U test, was used to compare responses from Q1 and Q2, please refer to Table A1 in the appendix. There was a statistically significant difference between the understanding of the MOTOM (*p* < 0.001) and where the MOTOM sits within the wider assessment (*p* < 0.001) between Q1 and Q2 respondents. Q1 respondents reported having a better understanding for both statements. For Q1 94.4% and 90.5% of respondents ‘agreed’ or ‘strongly agreed’ to the statements, respectively, compared to 70.1% and 78.8% for Q2 respondents. This better understanding is what we may intuitively expect as Q1 staff write or authorise the assessment whereas Q2 staff do not.

#### Qualitative findings

The following themes emerged from the open-ended questions within Q1 and Q2 and will be discussed.

##### The MOTOM is a service user tool

The MOTOM can be a benefit to the service user as it can allow for them to identify their own reablement goals.

“This approach is person-centred allowing the goals to be reflective of the service user and not from a specific list” (Q1R54).

The staff felt the most common goal set using the MOTOM was self-care related. A large proportion of staff felt this was the case because self-care was more important to the service user than productivity or leisure goals and was therefore reflective of their wishes.

“. . .a change in capacity to manage [self-care] is immediately apparent and of higher concern to service users” (Q1R25).

In addition, self-care may be more important to the individual because it helps to maintain their dignity. This shows how the MOTOM may improve outcomes other than independence, such as improvement in well-being.

“[service users] want to regain their independence . . . to protect privacy and dignity” (Q1R54).

The MOTOM facilitates goal setting; however, the goals may need to be reviewed. Staff felt that self-care goals were set initially due to these being a higher concern for service users. Respondents identified that once these goals have been achieved, the service user may have further goals they wish to work on. It may be difficult for reablement services to facilitate this due to time and capacity pressures.

“Mainly due to time and pressure of the service” (Q2R28).

However, the MOTOM could be used as a tool for service users to create short-term and long-term goals.

“[Self-care] is usually the first part . . . before moving onto other tasks” (Q2R112).

##### The MOTOM is a service provider tool

Although the MOTOM can be a service user tool, the scoring is not always shared with the service user and may be more of a benefit to the service provider. The staff explained that the MOTOM scores can support them within their roles as it provides an overview of the service user’s abilities and facilitates multidisciplinary team working.

“Any health professional within [the service] can come in look at the scores and see where the service user is progressing. . .” (Q2R108).

In addition, some of the goals set using the MOTOM appear to be based off the assessment by the professional and their own observation of a service user.

“Most common goals are around washing and dressing, as mainly identified as a need by RSW’s” (Q1R7).

The data showed that staff worked within a perceived service remit that could impact the types of goals set. This may be because reablement staff are aiming to prevent, reduce or delay the likelihood of a service user needing an ongoing package of care. As a result, the reablement staff may target certain activities of daily living to reduce potential ongoing care and make reablement a cost-effective service.

“We don’t do housework or assist [service users] to complete leisure activities such as accessing the community” (Q1R43).

However, not all staff appeared to share the same understanding of the service remit which could cause inconsistencies with goal setting.

“The OTs are encouraged to carryout holistic assessments alongside considering the specific referral request” (Q1R55).

##### The MOTOM provides quality assurance

A further benefit to the MOTOM was that it provided a framework for staff to use. Respondents clearly felt the MOTOM provided a structure for their assessment.

“It sets a start point and send point for the whole reablement. . . “(Q2R75).

Staff also reflected on how the MOTOM allows them to monitor an individual’s progression. The scoring of reablement goals provided evidence and can show a change in occupational performance.

“It does demonstrate a change in presentation as an outcome measure, and provides evidence of the intervention/outcome” (Q1R18).

Another way the MOTOM provided quality assurance was because it could be used by all reablement staff which helps in a large service.

“All of the people involved as well as the user work toward the same goal” (Q2R35).

##### The MOTOM can have restricted applicability

A limitation of the MOTOM was that it has restricted applicability and does not work for every individual. Reablement staff reflected on how there were many cases when goal setting was not appropriate for people due to their situation, for example within palliative care.

“Not everyone is able to reable” (Q1R27).

Certain staff also reported that goal setting can sometimes be unfavourable from a service users’ point of view.

“I think some aspects can seem invasive or sometimes childish” (Q1R14).

The MOTOM is being used within a time-sensitive setting and therefore, this could reduce the goal setting scope.

“. . .need[s] to be something you can work on directly and improve in a short space of time” (Q1R4).

Furthermore, the MOTOM may not provide the context behind the reablement intervention. Staff members reflected how the MOTOM could not be used as a stand-alone assessment.

“Sometimes reablement is more complex than what the MOTOM will show” (Q2R59).

##### Increased assessment training is required

Although the quantitative findings showed majority of staff felt they understood the MOTOM, the open-ended responses did not fully reflect this. Respondents felt that there needed to be further training provided when initially starting their role.

“Training in the paperwork side is lacking when initially starting out” (Q2R25).

Staff also felt that there was inconsistency within goal scoring between other members of staff, which reduces the usefulness of the MOTOM.

“Not all staff grade it entirely the same way meaning scores vary” (Q1R14).

## Discussion and Implications

In this section, the quantitative and qualitative findings will be discussed to assess if the MOTOM is an appropriate tool for reablement. Strengths and limitations of the MOTOM will be considered in relation to goal setting, goal scoring, person-centredness and training.

### Goal setting

One of the MOTOM’s key strengths is that it is a tool for goal setting (James and Coor, 2004). The quantitative findings of this research highlight that most staff agreed ‘that the ‘MOTOM allows for service users’ own goals to be formulated’. The qualitative findings revealed that goal setting is a benefit to both the service provider and the service user. Goal setting is at the centre of reablement, and some have argued that it’s ‘undeniably its most essential feature’ (Coltworthy and Westendorp, 2023: 225). It is also recognised that occupational therapists contain strengths with regards to goal setting due to their understanding of the impact of disability and injury (SCIE, 2011). In addition, the MOTOM is flexible with regards to what types of goals can be created as the tool does not have pre-determined categories. This enables service users to work on what matters most to them which is a strength of the tool (Azim et al., 2022).

To enable effective goal setting, there needs to be a clear understanding of the service criteria prior to the use of a goal-setting tool. The quantitative findings showed that leisure goals were the least common type of goal. In the findings, there appears to be slight confusion between staff members about the service criteria, with some staff feeling they were able to support service users with leisure activities, whereas others felt they were not. As a result, staff may restrict the types of goals they set with the service user to work within this perceived criterion. Consequently, the high proportions of self-care goals identified within this research may not be because of the MOTOM itself, but the understanding of the criteria of the reablement service. The NICE guidelines state services need to ‘recognise that participation in social and leisure activities are legitimate goals of intermediate care’ (NICE, 2017: 30). Previous research has also acknowledged that reablement may focus on self-care (Coltworthy and Westendorp, 2023). Therefore, the understanding of the reablement service criteria may negatively impact the MOTOM’s ability to be an effective reablement tool.

Another reason for the focus on self-care goals may be due to the service users understanding of the reablement criteria, which can impact goal setting (Rostgaard et al., 2023). Service users should be informed about how reablement can support them to reconnect with other goals such as productivity and leisure. Research has shown that service users ‘did not take an active role’ within goal setting (Jokstad et al., 2020: 1085). Therefore, for the MOTOM to facilitate successful goal setting, the service remit needs to be explained to service users to enable them to have an active role in this process.

A further finding of this research is that service providers and service users may prioritise certain goals. Service users may prioritise self-care goals initially, as individuals need to meet their physiological needs and feel safe and secure until they are able to progress (Maslow, 1943). NICE guidelines (2017) suggest that regular assessments should be completed, and goals should be developed throughout the reablement trajectory (Rostgaard et al, 2023). A strength of the MOTOM is that it requires the goals to be reviewed post-intervention. During this review, new goals could be created and as a result, the MOTOM permits both short-term and long-term goal generation. However, in this research, it is not clear whether new goals are being identified during the review. This could cause productivity and leisure goals to be neglected as these are likely to not be prioritised at the service user’s initial assessment.

### Goal scoring

Another strength of the MOTOM is that it provides a pre-outcome and post-outcome score and as seen from the qualitative findings, this enables the service provider to establish if certain interventions have been successful which has shown to be critical to the success of reablement (Tuntland et al, 2023). The Social Care Institute for Excellence advises for reablement that the ‘focus should be on achieving outcomes rather than completing care tasks’ (SCIE, 2020), this is a benefit of the MOTOM.

Although the scoring is a strength of the MOTOM, this research has highlighted that the implementation of the scoring can be subjective. The variation in the scoring may be due to several factors such as the professional’s different interpretation of independence. In addition, the scoring may fluctuate depending on the service user’s condition and as a result their independence may be variable, making it hard for the professional to apply one rating. If the MOTOM lacks reliability, the outcome scores will lose meaning and the tool will not be useful. James and Coor (2004) advised that the reliability of the MOTOM has not been formally assessed. For the MOTOM to be an appropriate reablement tool and to overcome some discrepancies in scoring, there is a need for consistent and regular training if staff confidence and reliability is to be maintained within the service.

### Person-centredness

Within this study, the MOTOM was used with every service user. Although the MOTOM contributes to a person-centred assessment, the findings showed staff felt the MOTOM does not work for everyone. This finding is consistent with other research that states ‘reablement has been criticised for being a “one size fits all” intervention’ (Coltworthy and Westendorp, 2023: 229). The NICE guidelines states reablement should be ‘tailored to the person’. To enable reablement to be person-centred, there may be times when the MOTOM is not appropriate to use. In addition, this local authority did not utilise the outcome codes as suggested in the manual and may benefit from doing so, for example outcome code ‘F’ is for ‘patient declines input’ or ‘U’ for ‘patient is too unwell’ (James and Coor, 2006: 9–10).

Furthermore, in this reablement service, the assessments are sent to the service user. The quantitative findings in this research have shown 52.8% of Q1 respondents and 41.4% for Q2 respondents agreed that ‘service users understand their written copy of the assessment’, suggesting a large proportion of staff have some doubt as to whether the service users understand the assessment. It is therefore important to consider what may need to be adjusted to make this more useful.

Although the reablement goals should be identified by the service user, it is not clear whether the scoring of the reablement goals within this setting are being applied solely by the professional or whether they are set collaboratively with the service user. James and Coor (2004: 213) report ‘while MOTOM is a patient-centred measure, it is not patient led’. This could be researched further with service users to consider if this would facilitate increased person-centred care.

### Training

The quantitative findings showed that Q1 staff’s confidence administering the MOTOM reduced for respondents that had been in the service for over 5 years. Additionally, for Q2 respondents, the level to which they agreed that they understood the MOTOM also reduced for staff that had been in the service 4–5 years. James and Coor (2004) investigated staff’s perceptions of the MOTOM in alternative settings to reablement and found that there was a need for regular updates and the use of case studies in training. If confidence and understanding increased, the MOTOM will be implemented more successfully.

## Limitations

There are a few limitations of this research. This local authority used the MOTOM in conjunction with its own reablement assessment form. It is important that we consider some of the findings may be conflated with the whole reablement assessment process and not solely the staff member’s opinions on the MOTOM. In addition, the questionnaire had a response rate of 37.7%, and due to participation being voluntary, it is possible there is a self-selection bias within the results.

The research lead also continued to work part-time as a reablement OT within this service whilst completing the research project. Preventative steps were taken to reduce bias throughout this research including the research lead keeping a reflexive diary and regular meetings with a supervisor with a different professional background. Although steps were taken to reduce bias, mainly from the research team, it is difficult to fully eliminate this.

## Conclusions

This research has shown that the MOTOM has key strengths within a reablement context. The MOTOM can allow for service users’ own goals to be generated, the scoring allows for team working and it provides a form of quality assurance for staff. These strengths adhere to the NICE guidelines (2014). There are potential weaknesses to the MOTOM as it may not be appropriate for specific cases where goal setting can be difficult, to increase the applicability of the MOTOM and to develop this service, the outcome codes could be utilised. In addition, the MOTOM may not always capture an individual’s full reablement plan. Although this research has limitations, it has shown more understanding of the MOTOM and the service criteria is required for confident and consistent use by reablement practitioners. There is a need for regular and consistent training on the measure. Both of these factors need to be addressed before the MOTOM’s full merit can be assessed. Future research should investigate service users’ perspective and experience of the MOTOM to ensure a more complete and inclusive answer to the research question.

**Key findings**

- The MOTOM has benefits within a reablement setting such as goal setting, monitoring progression, team working and quality assurance.
- The MOTOM can have restricted applicability, and the service providers lack consistency in goal scoring.
- Further training is needed to improve the understanding of the service criteria to enable non self-care goal generation.

**What the study has added**

This study has investigated the use of the MOTOM in a community reablement setting, it has identified strengths and weaknesses of the outcome measure by surveying the staff who use it.

## Appendix

**Table A1.** Mann–Whitney *U* Test: Comparing Questionnaire 1 and 2.

Question	Questionnaire	Sum of ranks	*p* Value
I understand what the Morriston Occupational Therapy Outcome Measure (MOTOM) is.	Questionnaire 1	92.91	0.001*
	Questionnaire 2	66.59	
I understand where the MOTOM sits within the wider reablement assessment.	Questionnaire 1	89.22	0.001*
	Questionnaire 2	69.69	
The MOTOM is an effective tool for capturing reablement interventions and progress.	Questionnaire 1	81.04	0.325
	Questionnaire 2	74.80	
There is consistency across staff members in how the MOTOM is completed.	Questionnaire 1	68.23	0.053
	Questionnaire 2	81.78	
The most common types of goals set using the MOTOM.	Questionnaire 1	77.05	0.593
	Questionnaire 2	75.42	
The MOTOM allows for service users own goals to be formulated.	Questionnaire 1	78.28	0.672
	Questionnaire 2	75.55	
The service users understand their written copy of the assessment.	Questionnaire 1	80.30	0.387
	Questionnaire 2	74.46	
Is there anything you would improve, include or remove in the reablement assessment?	Questionnaire 1	76.73	0.417
	Questionnaire 2	71.71	

*N* = 153, **p* < 0.05.
